# Large Scale Laser Crystallization of Solution-based Alumina-doped Zinc Oxide (AZO) Nanoinks for Highly Transparent Conductive Electrode

**DOI:** 10.1038/srep15517

**Published:** 2015-10-30

**Authors:** Qiong Nian, Michael Callahan, Mojib Saei, David Look, Harry Efstathiadis, John Bailey, Gary J. Cheng

**Affiliations:** 1 Birck Nanotechnology Center and School of Industrial Engineering, Purdue University, West Lafayette, IN 47906; 2 Greentech Solutions Inc., Hanson, MA 02341; 3 Semiconductor Research Center, Wright State University, Dayton, OH 45435; 4 CNSE College of Nanoscale Science and Engineering, University of Albany, Albany, NY 12203.

## Abstract

A new method combining aqueous solution printing with UV Laser crystallization (UVLC) and post annealing is developed to deposit highly transparent and conductive Aluminum doped Zinc Oxide (AZO) films. This technique is able to rapidly produce large area AZO films with better structural and optoelectronic properties than most high vacuum deposition, suggesting a potential large-scale manufacturing technique. The optoelectronic performance improvement attributes to UVLC and forming gas annealing (FMG) induced grain boundary density decrease and electron traps passivation at grain boundaries. The physical model and computational simulation developed in this work could be applied to thermal treatment of many other metal oxide films.

AZO is currently under fast development and extensive application to replace indium tin oxide (ITO) as TCO film^1^, though latter one is the standard compound for most devices performing best optoelectronic property^2^. Since it is crucial that AZO ensure a sustainable supply of the earth abundant and cost-effective alternatives. This is reflected in predicted markets of $925 million in 2016 for alternative TCOs^3^. Meanwhile, AZO thin films also exhibit impressive and reliable optoelectronic performance and environmental benign^2^,^4^, leading to wide installment in smart windows^2^,^5^, screen displays, photovoltaic cells^6^,^7^, and other optoelectronic devices^8^. Various deposition techniques are investigated to prepare high quality AZO film with resistivity of 10^−3^ Ω cm and visible transmittance over 85%, such as DC sputtering^9^, pulsed laser deposition (PLD)^10^, Atomic layer deposition (ALD)^11^ and chemical vapor deposition (CVD)^12^. However, these high vacuum methods are accompanied with key issues like instrumental complexity, high investment costs and limits scalability^2^, thus there is an ever-increasing demand to develop low cost non-vacuum deposition, especially for cutting edge flexible printing electronics. In this scheme, interest in solution based coating/printing and post treatment has been centered in breakthrough in the low profile fabrications. However, to the author’s knowledge, AZO film with high electrical conductivity (~1000 S cm^−1^) with transparency over 85–90% (T@550 nm) on the basis of non-vacuum deposition has not been succeed yet.

In principal, AZO thin films drew incredible attention due to significant advantages such as chemical stability in reducing environments and moreover the stabilized electrical property by aluminum dopant, which brought free charge carriers and simultaneously impede the chemisorption of oxygen at surface and grain boundaries^13^. However, since these polycrystalline AZO thin film coatings contain extended defects like inter-grain voids, gaps and grain boundaries, serving as electro traps at grain boundaries being detrimental to overall optoelectronic performance^14^. For instance, these defects tend to bring free carrier scattering centers and decrease the carrier lifetime so as to drawback the carrier mobility, resulting in low electrical conductivity^15^. Although electrical conductivity can be enhanced by increasing impurity doping level, this is usually compensated at the expense of red/IR transparency^12^. Thus, carrier scattering center minimization, in low impurity doping level with low carrier concentration, become critical to optimize carrier mobility and achieve satisfied electrical conductivity, without any compromise on transparency.

In this study, the method of mediating aqueous solution grows and post UVLC was built to overcome the bottleneck. The AZO precursor paste was painted onto the Cole-Parmer glass and followed by UV Laser exposure to minimized the extended defects and improve the optoelectronic performance as shown in Fig. 1a. Nevertheless, in contrast with interests focusing on the crystallization process itself, reports on the crystallization mechanism is rare and only little attention has been paid to the basic physical model. Herein, these key issues are tackled and physical model is built, which could apply to other N type oxide semiconductor oxide film thermal treatment.

## Results and Discussions

**Figure 1:**
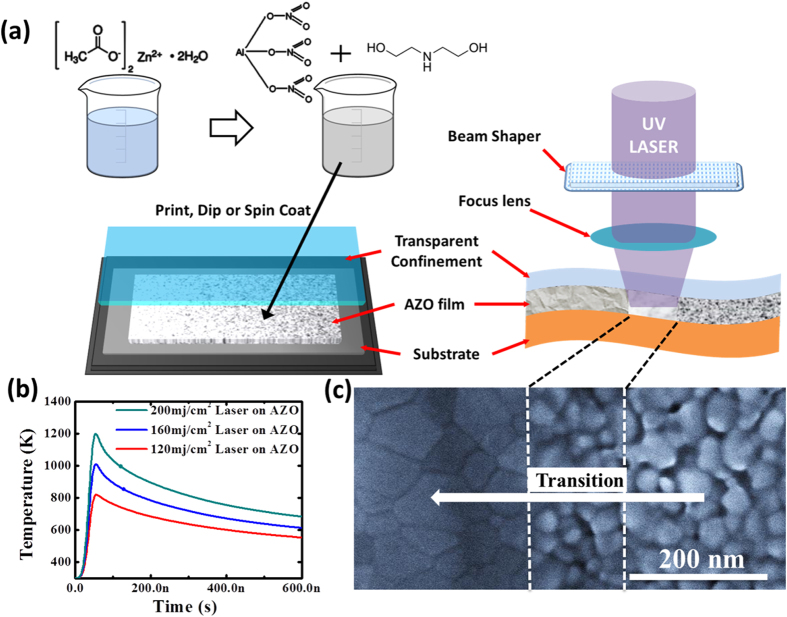
(**a**) The scheme of the AZO film aqueous solution grows and wet coating (left-hand side, potential wet coating includes: printing, dip coating and spin coating). And the scheme of post UVLC treatment on AZO film with Excimer laser and mirror systems (right-hand side); (**b**) The output of ComSol multiphysics® coupling module calculation: temperature evolution history of AZO film during UVLC treatment; (**c**) FESEM surface morphology observation of AZO film during UVLC processing.

Figure 1 show the scheme of the AZO film aqueous solution grows and the post UVLC treatment, associating with thermal distribution and temperature evolution simulated by COMSOL Multiphysics^®^. The aqueous solution grows based on chemical reaction between zinc acetate dihydrate and aluminum nitrate hexahydrate shown in Fig. 1a. Reacting precursor was coated to well-designed substrates, which can be quartz, sapphire, flexible glass or moderate melting point polymer according to demand. This wet synthesis activates various urgent coating techniques such as roll-to-roll printing, dip-coating and spin coating, boosting the scale-up ability and lowering the manufacturing cost massively. However, differentiating from high vacuum deposition, the wet synthesis of AZO nanofilm is likely to accompany with internal voids and gaps between particles, and oxygen species (oxygen and water) absorbed in grain boundaries serving as electro traps is inevitable. Industrial laser crystallization using large Excimer line lasers up to 0.75 M in length are used to convert amorphous silicon to high mobility polycrystalline silicon for the display industry^16^. Similar Excimer laser technology should be adaptable to printed metal oxide polycrystalline thin films to minimize internal imperfections and enhance optoelectronic performance. Thus, the pulsed Excimer Laser was scanning on the AZO film with shaped square beam and top-hat profile in size of 8 × 8 mm. The beam shape and size were modulated by adjusting beam shaper and the focus lens in Fig. 1a. Translations of substrate and scanning of laser beam was both enabled to boost the process efficiency and flexibility. During photo energy absorption, each Laser pulse was able to induce a localized high temperature field, because the band gap of AZO film is lower than the photo energy of Excimer Laser (*KrF Excimer Laser*, *5* *eV*)^2^,^4^,^17^.

Trying to understand the localized high temperature field generated by UVLC on AZO films, COMSOL Multiphysics^®^ was applied with electromagnetic module (EM) to simulate Laser irradiation, and the heat transfer module (HT) to describe the temperature increase on target film during a single Laser pulse delivery. Analyzing this temperature evolutionary history on AZO film would be beneficial to avoid overheating damage or film ablation^17^,^18^,^19^. Figure 1b plotting the output of this coupling module calculation, the temperature of AZO film increases to 800–1200 K in 60 ns depending on Laser fluences of 120–200 mJ cm^−2^, respectively. Then the temperature would be lowered by thermal dissipation, before subsequent multiple Laser pulse delivery. However, if thermal energy continues along multiple Laser pulse delivery, the accumulating heat drives crystallization process of AZO film. This is illustrated in Fig. 1c, the top view field emission scanning electron microscope (FESEM) image of a target film under processing, during which two neighboring particles start to melt over contacts area, then merge together and finally form large ones with apparent faceted boundaries. This interesting melting process over nanoparticle contacts area arouses the curiosity of the mechanism of heat generation between nanoparticles.

**Figure 2:**
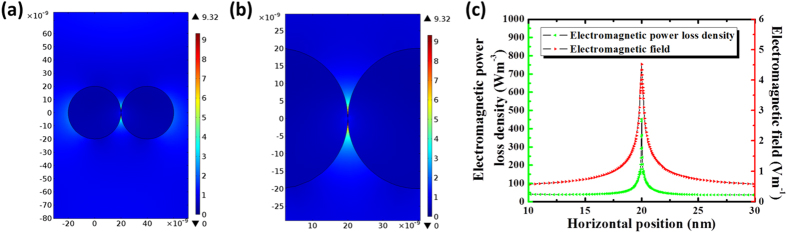
(**a**) In nanoscale, Laser-nanoparticle interaction simulated by ComSol Multiphysics simulation. Laser beam was simulated as an incident Gaussian electromagnetic wave with electrical field of 1 V/m on neighboring AZO nanoparticles. These two AZO nanoparticles were suspended in air and touching each other. Each nanoparticle was modeled as having a circular cross-section and essential physical parameters input. **(b)** The field distribution at magnified nanoparticle contacts. **(c)** The field distribution and heat generation in nano-scale as a function of distance to the contacts, described by power loss density calculated by Comsol Multiphysics^®^.

Since the temperature evolution history calculated by coupling module solely focuses on the macro scale crystallization effect on AZO nanoparticles film, while calculation of heat generation and mass transportation between nanoparticles during crystallization process are rare, thus deserve much attention. In nano-scale, laser-nanoparticle interaction, which is fundamentally different with bulk materials, needs to be aware of. As illustrated in Fig. 2a, two AZO nanoparticles are simulated with essential physical parameter impute, suspended in air and point contacted over surface, then exposed to ComSol Multiphysics simulated laser beam with electrical field of 1 V m^−1^. The bottom horizontal and left hand side vertical coordinates indicate the position of two neighboring AZO nanoparticles, while the color legends on the right hand side of the Figure exhibits local electrical field strength during laser-nanoparticle interaction. Unlike bulk material, the electrical field distribution of round shape nanoparticles is polarized due to plasmonic effect. The mechanism of plasmonic effect remains unknown, but it relates to enriched electrons and high relative permittivity of N-type semiconductor has been demonstrated in prior reports and our simulation. Polarized field distribution, magnified in Fig. 2b, shows intensive electrical field enhancement near nanoparticle contacts, achieving as high as 9 V m^−1^. While the field strength distributes inside nanoparticles remaining in lower level as high as 0 ~ 1 V m^−1^, indicating nanoparticle contacts form “hot spots” during laser-mater interaction. To further explore the heating process generated by the ‘hot spots’, heat generation of these ‘hot spots’ was determined from the illumination power density multiplies the AZO nanoparticle absorption coefficient (imaginary part of the dielectric function)^20^, that is, the power loss density calculated in Comsol Multiphysics^®^. The electromagnetic power loss density as a function of distance to nanoparticle contacts was plotted in Fig. 2c. The Heat generation decreases as distance to nanoparticle contacts increasing, implying the effective heat zone of initial laser scanning is constrained near contacts within around ±10 nm.

**Figure 3:**
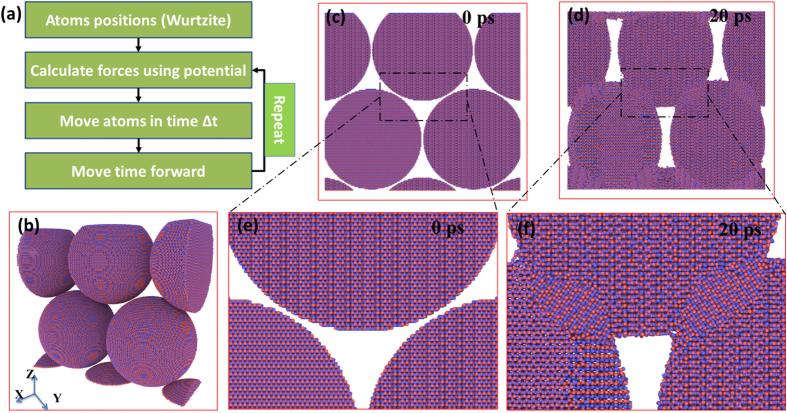
(**a**) MD simulation procedure of Laser heating on AZO nanoparticles stack based on nanoscale Laser-nanoparticle interaction. **(b)** Overall view of initial structure for half of the simulation box consisting of closely packed AZO particles plane through periodic boundary; the other half is another identical closely packed plane of particles with point contact between two planes. **(c)** Sliced plane cross front-view direction to observe AZO nanoparticles stack before UVLC; **(d)** after UVLC**. (e)** Focus-in view of crystal structure at contacts between AZO nanoparticles before UVLC; **(f)** after UVLC.

Although following heat dissipation and diffusion spread thermal energy all over nanoparticles and neighboring ones, the nanoparticle contacts still desire further investigation to determine how the heat generation and diffusion influence the phase transformation and mass transport during macro scale crystallization. Thus, Molecular Dynamic Simulation was performed to simulate Laser heating on three layers of closely packed AZO nanoparticles using LAMMPS^21^ with periodic boundary conditions applied in all three dimensions. The OVITO^22^ package was used for visualization. Firstly, the Laser heating input on these nanoparticles was set constrained in heat effect zone as calculated above, and then letting heat dissipation and diffusion spread all over. Secondly, to simulate atoms motion and mass transport between AZO nanoparticles, the standard Newton equations of motion were integrated in time using the velocity Verlet algorithm with a time step of 0.25 fs, as shown in Fig. 3a. The interaction between metal and oxygen atoms was modeled using the Binks potential (i.e. rigid ion approximation)^23^,^24^. The Binks inter-atomic potential has the form:


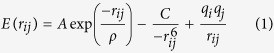


where *r*_*ij*_ is the distance between ions *i* and *j*, *q*_*i*_ and *q*_*j*_ are the electric charges of ion *i* and *j*. *Jason Binks* discussed the interatomic potential equation to reflect the effect of the electron cloud overlap and the dispersion. *A*, *ρ*, and *C* are potential parameters listed in Table 1. The parameters were also discussed by *Jason Binks*^23^,^24^, and they were chosen to reproduce the properties of the zinc oxide based lattice. On right-hand side of equation (1), the first and second terms describe the short-range repulsive and attractive interactions, respectively. The additional third term represents the long-range Columbic potential, which invokes the particle-mesh Ewald technique^25^. The cutoff for the Columbic term is set to 8 angstroms. Pairwise interactions within the cutoff distance are computed directly; interactions outside the cutoff distance are computed in reciprocal space.

Figure 3b only shows one plane of AZO nanoparticles consisted of 5 particles that touch each other in simulation box and through periodic boundary. Each particle with wurtzite structure is consisted of approximately 650000 atoms and 20 nm in diameter. However, overall three dimensional structures is closely packed in each plane and in point contact for out of the plane direction, two discrete planes touch each other on the poles of particles. Due to the memory limitations we only represent one plane of particles in the picture of Fig. 3b. Before the start of Laser heating part, equilibrium has been reached by running simulation in NPT ensemble for 10 ps. Laser heating has been applied to structure using a uniform heating all over the structure. To create hot spots, excessive heating has been applied over the connecting geometry of particles in a band of 30 nm in width. An exponential function has been used to identify excessive heating as a function of depth inward to touching particles. The uniform heating and the maximum in exponential heating are both equivalent to 5.6 μeV/fs per atom, meaning the maximum heating rate that an atom can acquire is 11.2 μeV/fs. Also this heating rate is very high but it is required for MD simulation to reach the same heating input as experiment in acceptable computing time. Figure 3c,d depict the structure modification of AZO particles stack before and after heating, which clearly show the particles coalesce together and forming “patch” like contact phase after UVLC. The new phase in state of gaps between particles, results from atom movement driven by Binks inter-atomic potential change during laser/heat-matter interaction. The formation of patch-like phases tends to connect particles, squeeze internal gaps and voids and finally condense loose AZO nanoparticles stack. The reducing of internal gaps and voids, which serve as mid-band electron traps, is crucial for electron mobility improvement. Moreover, as demonstrated by focus-in observations of Fig. 3e,f, the lattice structure formed by mass transport though contains plenty of disordering, tends to generate ordering alternatively arranged zinc atoms and oxygen atoms to certain extent. However, rather than ordering crystal lattice, even amorphous boundary between particles would definitely assist electron migration comparing with inter-particle gaps which are usually supposed as infinite potential well only allowing electron tunneling. On the other hand, the amorphous boundaries allow both electron tunneling and barrier crossing, while the latter one is dominant and determined by the barrier height which finally traces back to the disordering structures and electro traps at boundaries.

**Figure 4:**
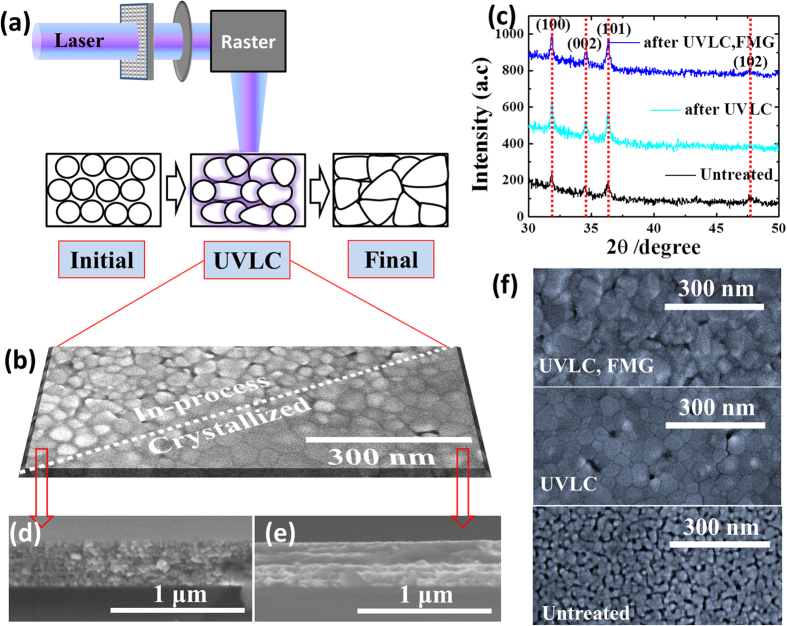
(**a**) Proposed mechanism scheme of UVLC process on AZO film. **(b)** Plane-view FESEM image of AZO film under ongoing UVLC. And cross-section FESEM image of AZO film before UVLC **(c)** and after UVLC **(d)**.

Both macro scale temperature evolution history of AZO film and the nanoscale atoms motion/mass transport of AZO nanoparticles stack during Laser heating, suggest the mechanism of UVLC. During UVLC, closely packed AZO nanoparticles film sustains three processes, as schemed in Fig. 4a. (1) Initial status of AZO nanoparticles stack was supposed as cubic close packing (ccp) right after wet coating and drying. (2) While during UVLC, the formed hot spots over contacts tend to drive small particles surface melting and merging together. This process tends to enlarge the grain size, modify the grain shape and compact internal gap. (3) The final status shows a much denser film stack, where faceted large grains were achieved and impinged on each other.

These three processes could be demonstrated by surface morphology change in Figs 1c and 4b. Comparing in-process to finally treated area (Fig. 4b), it is found that the crystallized film is more compact and continuous, suggesting the crystallinity of the AZO film has been significant enhanced^4^,^14^. The good film homogeneity and crystallinity result from the grain growth, shape change and reductions of internal defects including inter-crystal gaps and voids^17^. The crystallinity modification of AZO film is also verified by X-Ray Diffraction Patterns (XRD) as shown in Fig. 4c. Typical peaks located at 2θ = 31.9°, 34.6°, 36.4°, 47.6° are well indexed to Zinc Oxide crystal planes of (100), (002), (101) and (102). Comparing to signals before UVLC, it is clear seen that the three strong peaks of AZO film after UVLC achieve much higher intensity, implying enlarged grain size and textured crystal orientation. Further exploring of the structural modification on AZO film was characterized by FESEM images taken on cross-section-view before and after UVLC, respectively, in Fig. 4d,e. Figure 4d illustrates the untreated AZO film stack, apparently showing closely packed particle layers in cross-section but inhomogeneous and discontinuous surface^4^,^26^ with plenty of voids and gaps in plan-view observation (Fig. 4f). Figure 4e subject to the crystallized AZO films after UVLC, show that the small grains grew to large ones and became facetted with apparent grain boundaries, where homogeneous and continuous film quality was accomplished in plan-view image (Fig. 4f). It is deserved to know the results denoted as ‘UVLC, FMG’ in Fig. 4c and 4f correspond to processing conditions of FMG (Forming gas treatment under 400 °C, for 1 hour) after UVLC, which will be discussed later.

As a consequence, with small size grain merged and larger size grain formed, it is straightforward to draw that grain boundary density was decreased. On the other hand, since the grain shape changed to facetted and impinged on each other, the inter grain defects like voids, gaps and discontinuity decrease, which originally may create energy levels in the band gap that tend to trap the free electrons and decrease their lifetime^15^. Both lower grain boundary density and less electron traps at boundaries are able to diminish the grain boundary barrier scattering and boost the electron mobility cross boundaries, which contribute to or dominate the polycrystalline AZO film mobility^12^,^27^,^28^. Additionally, as presented in Fig. 4e, the 350 nm thick AZO film was coated layer by layer with Sol-Gel paste. While after UVLC (172 mJ cm^−2^), it was observed around 150 nm thick top layer of the AZO film was completely melted and crystallized. The bottom layer started to crystallize because of the thermal dissipation, but not completely. The electrical measurements would be carried out based on the 350 nm thin film characterization and 150 nm top layer calculation.

**Figure 5:**
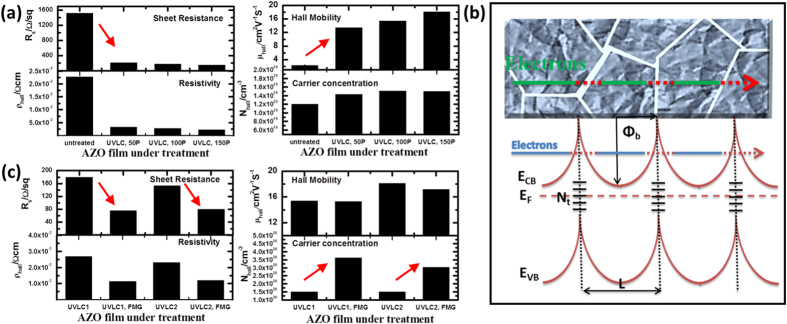
(**a**) Electrical properties of UVLC treated AZO films as a function of laser parameters, detected by Hall Effect Measurement; **(b)** Physical model of polycrystalline AZO film and energy potential barriers formed at grain boundaries; **(c)** Hall measurements of AZO films processed by UVLC with following FMG.

Figure 5a shows the electrical properties of UVLC treated AZO films as a function of laser parameters, detected by Hall Effect Measurement. After the crystallization, a strong decrease in sheet resistance R_s_ and resistivity ρ_hall_ is observed for all different Laser parameters. R_s_ decreases from 1.5 KΩ/sq to 217, 179 and 153 Ω/sq, respectively, when 50, 100 and 150 pulses were delivered to AZO film with fluence of 172 mJ cm^−2^. ρ_hall_ decreases from 2.28 × 10^−2^ Ω cm to 3.26 × 10^−3^, 2.69 × 10^−3^, 2.30 × 10^−3^ Ω cm in the same way. Both of them indicate the electrical conductivity has been significant enhanced with a factor of 10 times after UVLC. To further investigate the electrical conductivity enhancement, carrier concentration and carrier mobility of the untreated and processed films are plotted in Fig. 5a too. It is can be seen the conductivity enhancement associates with a strong increase in carrier mobility, as well as a moderate increase in carrier concentration. The mobility has been increased over 8 times after UV Laser treatment for the sol^−^gel paste fabricated AZO thin film. Under optimal Laser condition of 172 mJ cm^−2^ and 150 pulses, the solution based AZO thin film reach remarkably high mobility values of 18.1 cm^2^ V^−1^ s^−1^, simultaneously keeping a high carrier concentration of 1.50 × 10^20^ cm^−3^, and finally leading to resistivity as low as 2.30 × 10^−3^ Ω cm, which has not been succeed before to the author’s knowledge. Since carrier concentration in AZO thin film increase from 1.20 × 10^20^ cm^−3^ to 1.50 × 10^20^ cm^−3^ after UV Laser treatment, which could enhance conductivity by a factor of 1.25. This leave a conductivity enhancement factor of 8 resulting from carrier mobility increase, faying with measurements mentioned above^12^.

Moreover, according to *Delahoy et al.*^28^, free carrier mobility μ_hall_ of polycrystalline AZO film would be determined by four principal scattering process include ionized impurity scattering μ_i_^−1^, lattice vibration scattering μ_l_^−1^, grain boundary scattering μ_g_^−1^ and neutral impurity scattering μ_n_^−1^, as presented in equation (2). While the lattice vibration scattering μ_l_^−1^ could be neglected at room temperature and neutral impurity scattering μ_n_^−1^ is also negligible since the amount of neutral impurity scattering centers is much less than that of ionized impurity^29^. So the free carrier mobility μ_hall_ is mainly determined by ionized impurity scattering μ_i_^−1^ and grain boundary scattering μ_g_^−1^, as shown in equation (3).


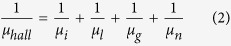



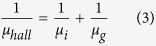


In polycrystalline conductive film like AZO, the intra-grain mobility (μ_i_) is usually dominated by ionized impurity scattering at room-temperature and grain boundary mobility (μ_g_) is usually determined by grain boundary scattering^12^,^27^,^28^,^30^. These two mechanisms compete with each other for constraining the total free carrier mobility referring to equation (3). For the relative low carrier concentrations (<2 × 10^20^ cm^−3^) in this study (1.2 − 1.5 × 10^20^ cm^−3^), it is generally agreed that grain boundary mobility is the bottleneck^28^,^31^. This means the hall mobility of the as-coated as well as processed AZO film is determined by the value of grain boundary mobility μ_g_ shown in equation (3). This also could be proved by μ_i_ value of 50–80 cm^2^ V^−1^ s^−1^ in current carrier concentration (1.2 − 1.5 × 10^20^ cm^−3^) calculated by Brooks-Herring-Dingle model^28^,^31^, which systematically summarized the previous experiment fittings^24^,^27^. However, taking into account of the combining effect in equation (3), the free carrier mobility μ_hall_ improvement in Fig. 2g after UVLC results from the vast grain boundary mobility μ_g_ increase from 2.42 to at least 25 cm^2^ V^−1^ s^−1^ with a enhance factor of 10.

To analyze the grain boundary mobility μ_g_ enhancement of AZO film after UVLC, the polycrystalline structure and energy level was schemed in Fig. 5b. Figure 5b shows free carriers (electrons in AZO) flowing in polycrystalline AZO film were scattered by grain boundaries. The grain boundary density is determined by grain size L while the scattering intensity at grain boundaries is determined by energy potential barrier height Φ_b_. The latter one is controlled by electron trap density (N_t_) and the free electron concentration (N_eff_). For all the thin films in current study, the product of the average grain size (~25-100  nm) times the electron concentration (1.2 × 10^20^ cm^−3^ − 1.5 × 10^20^ cm^−3^) is larger than N_t_ (<3.0 × 10^13^ cm^−2^), so that the grains are not fully depleted of the electrons^12^,^27^. Then first approximation can be applied to describe the energy potential barrier at grain boundary as shown in equation (4)^12^,^27^,^28^,^29^, in which 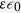 is the static dielectric constant and *e* is the elementary charge.


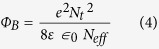



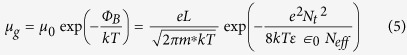


Considering both grain boundary density and energy potential barrier at grain boundaries, Seto and Baccarani^32^,^33^ extend Petriz model^34^ to describe the grain boundary mobility μ_g_ as shown in equation (5) where m^*^ is the electron effective mass, L is the grain size and Φ_b_ is the grain boundary potential barrier height. The basic result of this equation is based on electrons transport through grain boundary by thermionic emission over the barrier, taking into account of electron traps as a depletion region formed on either side of the grain boundary barrier. The average grain size L was increased by ~2 times (estimation) which leads to an enhance factor of 2 for the grain boundary mobility according to equation (5). However, as calculated from equation (2), the grain boundary mobility has been improved by 10 times which leave a factor of 5 attributing to the electron trap density N_t_ decrease at grain boundaries. The decrease of electron trap density attributes to removal of both extended defects (mid-band energy level) and desorption of oxygen species at grain boundaries^2^,^12^. The desorption of oxygen species could be achieved by UV annealing has been stated *Ding et al.*^12^, agreeing well with our UV laser exposure effect. Desorption of oxygen species would release free carriers from traps which is also stated before^2^,^12^. In addition, the UV exposure would dissociate Zn-O bond which results in oxygen vacancies formations, leading to free carrier concentration increase since oxygen vacancies acting as electron donors in AZO. Subject to current series of samples, these could be demonstrated by a moderate increase of carrier concentration after UVLC as shown in Fig. 5a. Formation of faceted grains removes extended defects such as inter-grain voids and gaps, making grains impinging on each other. This might change the Van der Waals boundaries at spherical nanoparticle contacts to tighter covalence grain boundaries.

Since carrier concentration in polycrystalline AZO film after UVLC still remain in low level (<2 × 10^20^ cm^−3^), impurity ion scattering still does not dominate in current series of sample, implying further increase in carrier concentration has a potential to enhance electrical conductivity more. As demonstrated in Fig. 5c, sheet resistance R_s_ of AZO thin film under UVLC1 (UV Laser crystallization with 172 mJ cm^−2^, 100 pulses) drops from 179 Ω/sq to 75 Ω/sq after FMG. Similarly, resistivity deceases from 2.69 × 10^−3^ Ω cm to 1.13 × 10^−3^ Ω cm. The sheet resistance drop and electrical resistivity decrease mainly due to the strong increase of carrier concentration from 1.51 × 10^20^ cm^−3^ to 3.61 × 10^20^ cm^−3^, because of free carriers release from FMG passivation oxygen species and other electron traps, without carrier mobility drawback. On the other hand, carrier mobility of AZO under UVLC2 (UV Laser crystallization with 172 mJ cm^−2^, 150 pulses) encounters a slight decrease from 18.1 to 17.3 cm^2^ V^−1^ s^−1^ after FMG, implying impurity ion scattering accumulate influence on carrier mobility. However, resulting from carrier concentration increase, a remarkable low sheet resistance of 79 Ω/sq is still achieved.

**Figure 6:**
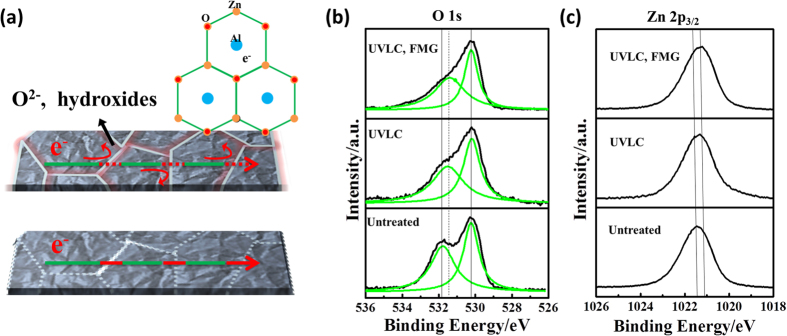
(**a**) Scheme of polycrystalline AZO and the electron traps formed by oxygen species at grain boundaries. The oxygen species tend to absorb electrons and form negative potential barrier. XPS spectra of AZO film before and after UVLC and FMG process **(b)** binding energy peak index to O 1s and **(c)** binding energy peak index to Zn 2p_3/2_. The green lines marked in **(b)** indicate deconvolution of O 1s spectra. **(d)** SEM surface morphology observation of AZO film before treatment, after UVLC process comparing with after UVLC/ FMG process. **(e)** XRD spectrum comparison between untreated AZO film and the ones after UVLC and UVLC/FMG processes.

FMG not only contributes to the release of free carriers but also desorption of oxygen species (electron traps) at grain boundaries, similarly to UVLC. This is crucial for film conductivity since the oxygen species (mainly oxygen and water) tends to absorb electrons to form insulation hydroxides phase, in which the absorbed electrons generate negative potential barrier at boundaries would be also detrimental for electron thermionic emission. This is illustrated in Fig. 6a. Desorption of oxygen species after UVLC and FMG can be further confirmed by XPS spectra as shown in Fig. 6b,c. As compared before and after UVLC, only a slight difference in the Zn2p_3/2_ signal was observed. And Al dopants were presented averagely uniform concentration in Secondary Ion Mass Spectrometry (SIMS, Figure S1), serving as electron dopant. In contrast, the O1s peak overall shape was affected dramatically after the UV laser exposure and FMG treatment. This O1s peak also could be deconvoluted (Lorentz fit) for further observation as marked by green lines in Fig. 6b. The deconvoluted peak located at higher binding energy (531 7eV) usually assigned to hydroxides or metal carbonates, it can be seen that the peak downshifted to lower energy typical for metal oxides, revealing oxygen chemical state affected. The FMG provide reactive N_2_/H_2_ during high temperature annealing, desorbed the oxygen species, leading to the formation of oxygen vacancies and passivation of electron traps at grain boundaries. The oxygen vacancies act as electron donors in film, thereby increase carrier concentration drastically, as reflected in Fig. 5c. In addition to desorption of oxygen species by FMG, the grain size change is also of great importance before and after FMG, which also might affecting electrons movement in polycrystalline film. Therefore, we took the FESEM images of AZO film before treatment, after UVLC and after UVLC/FMG process to monitor surface morphology change (Fig. 4f). We did not find apparent change of the grain size and shape induced by FMG. However, we clearly observed inter-grain gap increases slightly, which might be ascribed to thermal stress release and microstructure reorganization. So we intentionally collected the XRD spectrum to further confirm the grain size and surface morphology evaluation in (Fig. 4c). Based the XRD spectrum comparison with untreated sample, the grain size and texturing orientation are similar after UVLC process and after UVLC/FMG process, implying no apparent grain size parameter “L” change in equation (5), while the FMG process mainly contribute to electro traps “N_t_” decreases. The effect of UVLC and FMG on carrier concentration and mobility of current series of samples has been summarized and listed in Table 2. It should be aware that since the FMG treatment improves carrier concentration by desorbing oxygen species and forming oxygen vacancies. The treated AZO film should not be stored in ambient conditions since oxygen species like water might be able to absorb back to the film again and degrade the film conductivity. To avoid this issue, reduced pressure (lower than 10 mTorr) or protection atmosphere (nitrogen, argon) would be crucial for practical application.

**Figure 7:**
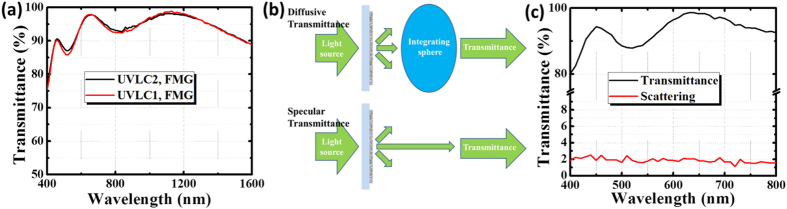
AZO film transmittance measurements. **(a)** Vis-IR transmittance spectrum of UVLC/FMG processed AZO films; **(b)** Light scattering measurement set up: The difference between the diffusive transmittance and the specular transmittance evaluates light scattering scale. **(c)** Light scattering spectrum of UVLC/FMG processed AZO film characterized by Vis-IR transmittance.

Not only electrical conductance but also transparency needs to be considered so as to evaluate the potential application of this solution fabricated AZO film like in practical touch screen display and smart window. UV-Vis-IR transmittance measurement was carried out to explore this optoelectronic performance. As illustrated in Fig. 7a and S3, the transmittance spectrum of the current series of samples in the wavelength range of 400–1600 nm were measured with Cole-Pamer glass as reference substrate. All results meet the requirements of touch screen display for practical application (R_s_: 500 Ω/sq; T: 85%). For instance, the films processed by 172 mJ cm^−2^ reach 75 Ω/sq with 88% T@550 nm and 79 Ω/sq with 89% T@550 nm (Fig. 7a, UVLC and FMG processed), depending on Laser pulse number and FMG, respectively. However, the same batch film processed by 172 mJ cm^−2^ reaches 217 Ω/sq with 95% T@550 nm without FMG treatment (Figure S3, only UVLC processed). To date, no such low sheet resistance (R_s_ < 80 Ω/sq @ T > 88%) has been reported before for the thorough solution fabricated AZO film, though combined with post treatment. This remarkable optoelectronic performance mainly attributes to the relaxing conductivity/transparency trade-off by increasing the charge carrier mobility after UVLC^2^,^12^. On the other hand, the UV-Vis-IR transmittance exhibits a slight decrease near Vis-IR range after FMG (Figure S3). Especially in visible range of 450–600 nm, the FMG processed samples show apparent lower transmittance owing to free carrier absorption, which is reflected in the moderate carrier concentration increase in Figure S3. As well as near IR range of 750–900 nm, a slight decrease of transmittance is also observed. These findings confirmed the statement of carrier release and desorption of surface oxygen. Under laser intensity of 192 mJ cm^−2^, the films reach 273 Ω/sq with 96% T@550 nm, 217 Ω/sq with 95% T@550 nm (Figure S3, only UVLC processed) and 95 Ω/sq with 89.4% T@550 nm (Figure S3, UVLC and FMG processed), depending on different laser pulse number and FMG, respectively.

Figure 7b shows the film scattered transmittance scale evaluation setup. The light scattering was quantified by the difference between diffusive and specular transmittance (the light comes out of the sample parallel to the incident light). The diffusive transmittance was measured by an integrating sphere to integrate all forward light including both scattered and specular transmitted light. After the baseline for the UV-Vis-IR spectroscopy was set by scanning a blank glass substrate, the AZO film was installed on a solid sample holder between light source and the detector for measurement. The quantification of scattering would be required, as well as comparison to other alternatives, due to crucial role in customer experience if manufactured in optoelectronic devices like touch screens and smart windows. As illustrated in Fig. 7c, the current series of AZO film (UVLC and FMG processed) exhibit ultra-low scattering transmittance in the full visible range (400–800 nm), implying macro-scale uniform and homogeneous film surface. Table 3 shows the diffusive and specular transmittance of AZO film measured by UV-Vis-IR spectroscopy at 550 nm wavelength in the same way. The difference between them are shown and compared with other transparent electrode alternatives^35^,^36^. It is can be seen that the scattering of AZO film is ~1.8% after UVLC and FMG. Comparing with other alternatives like CNT (~3.89%), silver nanowires (~11%) and Graphene hybrid film (h3, ~6.6%), whose high light scattering scale might trigger problematic for certain displays like touch screens^35^, the processed AZO film achieve significant low light scattering and lead to potential capability to compete with ITO (~1%)^35^,^36^.

Comparing aqueous solution fabrication in our work with other groups, the UV Laser crystallized AZO films exhibit high mobility (18.1 cm^2^ V^−1^ s^−1^), implying diminishing grain boundary barrier and decreasing grain boundary density. When carrier density was increased to ~3 × 10^20^ cm^−3^ by FMG, the UV Laser crystallized AZO film obtains slightly decreased carrier mobility, indicating the charge mobility dominated by both impurity ion scattering and grain boundary barrier scattering. The highest electrical conductivity of current series of sample reaches ~1000 S cm^−1^, which performs better than many vacuum methods. In addition, grain boundary density also could be affected by film thickness which would further influence the carrier mobility^12^. According to equation (4), the grain boundary density influences the grain boundary mobility with a linear factor. This supply an explanation that charge mobility in our study is still lower than some high vacuum fabrications like FCAD and Magnetic assisted PLD. However, considering the 150 nm thick AZO film in our work is extremely thinner than most prior advancements (μm scale), UV laser crystallization has potential to achieve even higher carrier mobility with the efficient low cost aqueous solution fabrication.

In summary, this new concept combined aqueous solution fabrication and post UVLC has been built to deposit transparent and conductive AZO films with better structural and optoelectronics properties than most high temperature/high vacuum deposition methods, suggesting a potential for low cost and large scale manufacturing. Under optimal UVLC parameter and FMG, an AZO film with low resistivity of ~1 × 10^−3^ Ω cm, high Hall mobility of 18.1 cm^2^ V^−1^ s^−1^, and low sheet resistance of 75 Ω/sq was obtained. Especially, the low sheet resistance was achieved with retaining high transmittance of 88%–96% @550 nm. The best result of current study with 75 Ω/sq and 88% T@550 nm has not been reported before for thorough aqueous solution fabrications and post treatments. The light scattering of the AZO film exhibiting just 1.8% also suggests a potential to compete with market mainstream ITO film. The outstanding optoelectronic performance mainly attributes to the grain boundary density decrease and the electron trap density decrease at grain boundaries.

## Methods

To synthesis AZO thin film, the precursor solution consisted of semiconductor grade ethanol in which zinc acetate dihydrate [Zn(CH_3_COO)_2_•2H_2_O] (0.4 M) was dissolved. Aluminum nitrate hexahydrate [Al(NO_3_)_3_•6H_2_O] was dissolved in an amount to yield 2% Al in relation to Zn. Diethanolamine [NH(CH_2_CH_2_OH)_2_, DEA] (1M) was added to the zinc acetate. The solution was heated to approximately 75 °C and stirred for 2 hours and allowed to cool to room temperature.

Then, soda lime glass slides were used as substrates for precursor coating. The slides were sectioned into pieces each with approximately 2.5 cm^2^ area. These pieces were then cleaned with deionized water and isopropanol and dried using an air gun with purified air. These substrates, one at a time, were then loaded into a Laurell WS-650 spin coater with an automatic dispenser unit. The precursor solution was dispensed onto each substrate 8 successive times with the substrate spinning at 500 rpm for dispensation and then 3000 rpm for drying. After each layer dispensed, it was evaporated and annealed on a hot plate at approximately 475 °C to convert each precursor layer to AZO. After all 8 layers achieved, the substrates were placed in a tube furnace and heated in argon with 2% hydrogen gas for 2 hours.

Then fabricated AZO thin film was put into a Nitrogen purged chamber for the UVLC process. KrF excimer laser (*λ* of 248 nm and *τ* of 25 ns) with repetition rate (RR) of 10 Hz was utilized in this study. The Laser beam was shaped to a square, top-hat profile (8 × 8 mm). In order to process large scale, the sample was placed on a motorized stage which enables translations along both X and Y axes. Laser intensities used in the crystallization experiments ranged from 130 to 210 mJ cm^−2^. The laser pulse number (*N*) used ranged from 50 to 150, corresponding to Laser shining time of 1.25–3.75 μs.

After the Laser crystallization, field emission scanning electron microscopy (FE-SEM) was used to observe the surface morphology and cross-section structure. The crystal size histograms can be measured from the top-view Fe-SEM images. Electrical resistivity, carrier mobility and carrier concentration were measured by the Hall Effect with the Van der Pauw method. Optical transmittance spectra were measured by Lambda 950 ultraviolet-visible and infrared (UV-Vis-IR) spectrophotometers.

## Additional Information

**How to cite this article**: Nian, Q. *et al.* Large Scale Laser Crystallization of Solution-based Alumina-doped Zinc Oxide (AZO) Nanoinks for Highly Transparent Conductive Electrode. *Sci. Rep.* **5**, 15517; doi: 10.1038/srep15517 (2015).

## Supplementary Material

Supplementary Information

## Figures and Tables

**Table 1:** Parameters for interatomic potential of MD simulation^23^,^24^.

Species		A(eV)	Ρ(Å)	C(eVÅ6)
O^2−^	O^2−^	9547.96	0.21916	32.0
Zn^2+^	O^2−^	529.70	0.3581	0.0
Zn^2+^	Zn^2+^	0.0	0.0	0.0

**Table 2:** effect of UVLC and FMG on carrier mobility dominants.

Carrier concentration	Carrier mobility dominant	Physical parameter	UVLC modification	FMG modification
>2 × 10^20^ cm^−3^	Ionized impurity scattering	doping level	No	No
		Carrier concentration	slight	Yes
<2 × 10^20^ cm^−3^	Grain boundary scattering	Grain boundary density	Yes	No
		Barrier height	Yes	Slight

**Table 3:** Performance of diffusive transmittance, specular transmittance and light scattering @550 nm of UVLC/FMG processed AZO films, comparing with glass, CNT, silver nanowires, and graphene hybrid material.

Transmittance @550 nm	Glass	AZO, UVLC/FMG	CNT	Ag Nanowire	Graphene Hrbid-3
Diffusive/%	91.5	88	75	79	88
Specular/%	90.02	86.2	71.11	68	81.4
HAZE/%	1.48	1.8	3.89	11	6.6
