# Supplementary material for: Large Scale Laser Crystallization of Solution-based Alumina-doped Zinc Oxide (AZO) Nanoinks for Highly Transparent Conductive Electrode
Source: Sci Rep. 2015 Oct 30;5:15517. doi: 10.1038/srep15517 (PMC4626788; doi:10.1038/srep15517)
Supplement: Supplementary Information [file srep15517-s1.doc]

**Large Scale Laser Crystallization of Solution-based Alumina-doped Zinc Oxide (AZO) Nanoinks for Highly Transparent Conductive Electrode**

*Qiong Nian^1^, Michael Callahan^2^, Mojib Saei^1^, David Look^3^, Harry Efstathiadis^4^, John Bailey^2^, Gary J. Cheng^1^**

*1. Birck Nanotechnology Center and School of Industrial Engineering, Purdue University, West Lafayette, IN 47906*

*2. Greentech Solutions Inc., Hanson, MA 02341*

*3. Semiconductor Research Center, Wright State University, Dayton, OH 45435*

*4. CNSE College of Nanoscale Science and Engineering, University of Albany, Albany, NY 12203*


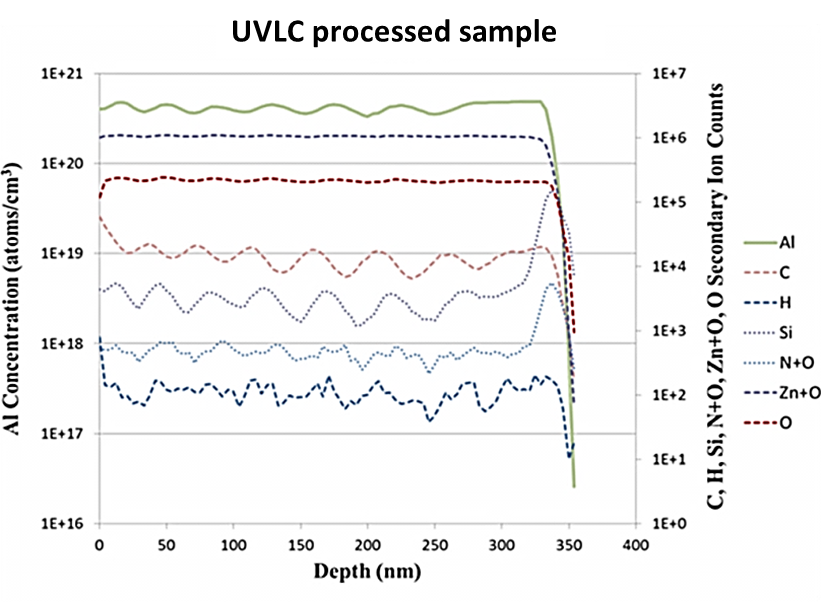


**Figure S1** Secondary Ion Mass Spectrometry spectrum of AZO film processed after UVLC process.

Secondary Ion Mass Spectrometry (SIMS was performed) of the UVLC processed AZO film is shown in S3. Two pertinent insights came from the results. Firstly, carbon concentration in the AZO films is 2 orders of magnitude lower than the Al concentration and thus should not negatively impact mobility. The carbon incorporation was a direct result of inorganic solvents used for spin coating. This is significant as carbon base inks are preferable than water base inks. Secondly, the SIMS clearly shows that the chemical profile of the sol-gel layers is retained during the whole UVLC process.


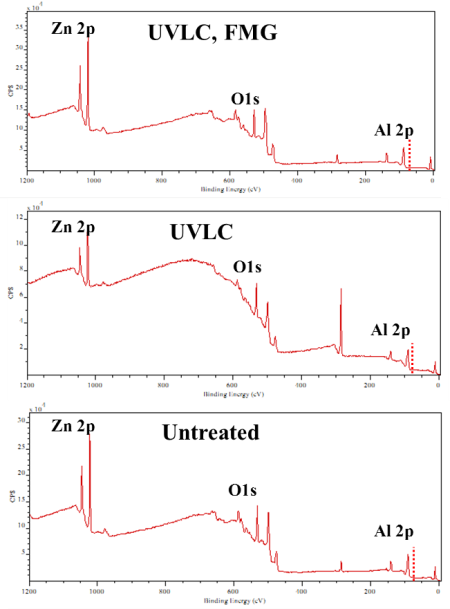


**Figure S2** Full XPS spectrum of AZO sample under three different process conditions: untreated, UVLC processed, and UVLC/FMG processed.

Combining with figure S1, manuscript figure 6, this supporting figure S2 further confirmes desorption of oxygen species after UVLC and FMG can. The reseaon is, as compared before and after UVLC, only a slight difference in the Zn2p3/2 signal was observed in manuscript figure 6, implying negligible change of zinc phase and ignorbale performance effect of zinc. As well as Al2p, which is important to analyze due to moderate effect of aluminum phase on film microsctructure and performance. Therefore, figure s1 (Secondary Ion Mass Spectrometry spectrum) and figure S2 (full XPS spectra) were taken to monitor aluminum phase, in which no apparent change of Al ratio and phase were observed. In contrast, the O1s signal was affected dramatically after the UV Laser exposure, where the overal peak shape has been dramatically changed. And the deconvoluted peak at higher binding energy (located at 531.7eV) usually assigned to hydroxides and metal carbonates downshifted to lower energy typical for metal oxides.


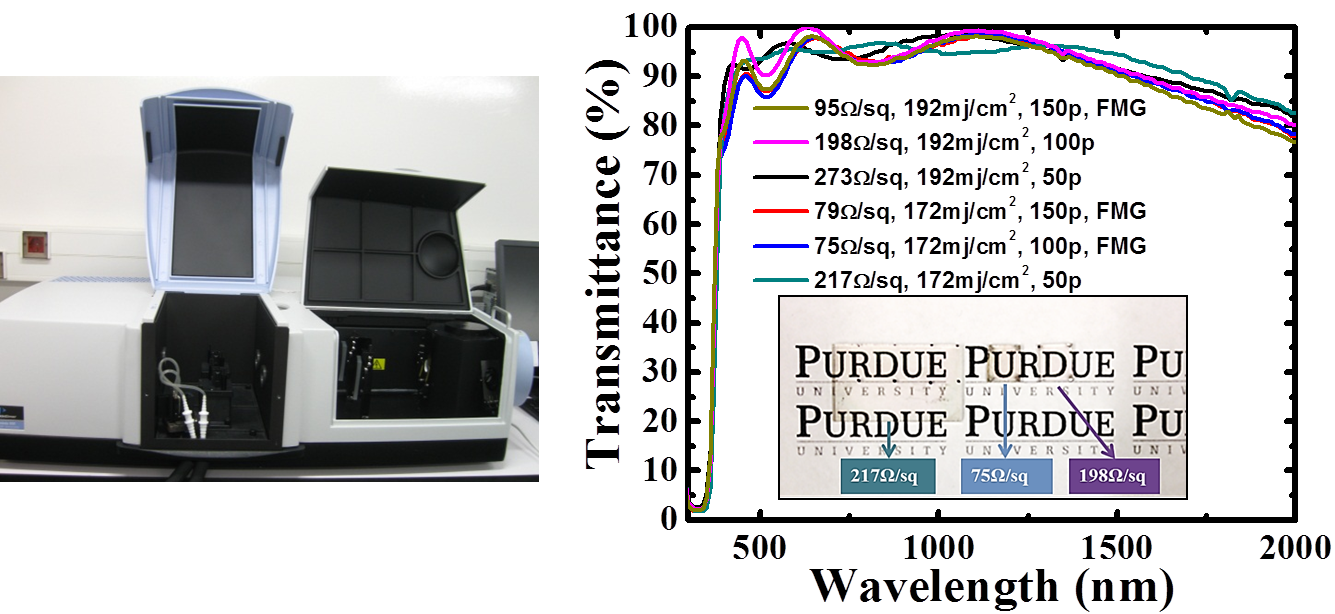


**Figure S3** UV-Vis-IR transmittance measurement was carried out to explore this optoelectronic performance. Left-hand-side figure shows the equipment of lambda 950. The-right-hand side shows the transmittance spectrum of the current series of samples (after UVLC and after UVLC/FMG process) in the wavelength range of 300-2000 nm were measured with Cole-Pamer glass as reference substrate.

Figure S3 show all transparence measurement results after UVLC and UVLC/FMG process meet the requirements of touch screen display for practical application (R_s_: 500 Ω/sq; T: 85%). For instance, the films processed by 172 mJ cm^-2^ reach 217 Ω/sq with 95% T@550nm (only UVLC processed), 75 Ω/sq with 88% T@550nm and 79 Ω/sq with 89% T@550nm (UVLC/FMG processed), depending on Laser pulse number and FMG, respectively. To date, no such low sheet esistance (R_s_<80 Ω/sq @ T >88%) has been reported before for the thorough solution fabricated AZO film, though combined with post treatment. This remarkable optoelectronic performance mainly attributes to the relaxing conductivity/transparency trade-off by increasing the charge carrier mobility after UVLC. As well as laser intensity of 192 mJ cm^-2^, the films reach 273 Ω/sq with 96% T@550nm, 217 Ω/sq with 95% T@550nm (only UVLC processed) and 95 Ω/sq with 89.4% T@550nm (UVLC/FMG processed), depending on different Laser pulse number and FMG, respectively. On the other hand, the UV-Vis-IR transmittance exhibits a slight decrease near Vis-IR range after FMG owing to free carrier absorption, which is reflected in the moderate carrier concentration increase in manuscript figure 5. As measured, after FMG process, due to chemical reaction between annealing forming gas and absorbed oxygen species, extra carriers were released thereby increasing from ~1.5×10^20^ cm^-3^ to 3.6×10^20^ cm^-3^.

**Reference:**

1 Ding, L., Nicolay, S., Steinhauser, J., Kroll, U. & Ballif, C. Relaxing the Conductivity/Transparency Trade-Off in MOCVD ZnO Thin Films by Hydrogen Plasma. *Adv. Funct. Mater.* **23**, 5177-5182, doi:10.1002/adfm.201203541 (2013).

2 Hagendorfer, H. *et al.* Highly Transparent and Conductive ZnO: Al Thin Films from a Low Temperature Aqueous Solution Approach. *Adv. Mater.* **26**, 632-636, doi:10.1002/adma.201303186 (2014).
